# Viral meningoencephalitis in pediatric solid organ or hematopoietic cell transplant recipients: a diagnostic and therapeutic approach

**DOI:** 10.3389/fped.2024.1259088

**Published:** 2024-02-12

**Authors:** Sanya J. Thomas, Christopher P. Ouellette

**Affiliations:** ^1^ Host Defense Program, Section of Infectious Diseases, Nationwide Children’s Hospital, Columbus, OH, United States; ^2^ Division of Infectious Diseases, Department of Pediatrics, Ohio State University College of Medicine, Columbus, OH, United States

**Keywords:** encephalitis, viral, pediatric, transplantation, immunocompromised

## Abstract

Neurologic complications, both infectious and non-infectious, are frequent among hematopoietic cell transplant (HCT) and solid organ transplant (SOT) recipients. Up to 46% of HCT and 50% of SOT recipients experience a neurological complication, including cerebrovascular accidents, drug toxicities, as well as infections. Defects in innate, adaptive, and humoral immune function among transplant recipients predispose to opportunistic infections, including central nervous system (CNS) disease. CNS infections remain uncommon overall amongst HCT and SOT recipients, compromising approximately 1% of total cases among adult patients. Given the relatively lower number of pediatric transplant recipients, the incidence of CNS disease amongst in this population remains unknown. Although infections comprise a small percentage of the neurological complications that occur post-transplant, the associated morbidity and mortality in an immunosuppressed state makes it imperative to promptly evaluate and aggressively treat a pediatric transplant patient with suspicion for viral meningoencephalitis. This manuscript guides the reader through a broad infectious and non-infectious diagnostic differential in a transplant recipient presenting with altered mentation and fever and thereafter, elaborates on diagnostics and management of viral meningoencephalitis. Hypothetical SOT and HCT patient cases have also been constructed to illustrate the diagnostic and management process in select viral etiologies. Given the unique risk for various opportunistic viral infections resulting in CNS disease among transplant recipients, the manuscript will provide a contemporary review of the epidemiology, risk factors, diagnosis, and management of viral meningoencephalitis in these patients

## Introduction

Up to 46% of children who undergo hematopoietic cell transplantation (HCT) and 30%–50% pediatric solid organ transplant recipients experience neurological complications (Table 1). Among HCT recipients who experience neurological complications, a lower incidence reported in the first year post-transplant compared to later time points (1–6). In an Israeli study spanning two decades and including more than 700 pediatric HCT recipients, non-infectious complications were found to be more common (81.3%) compared to infectious complications (18.7%) (7). Factors commonly associated with increased risk of neurological complications included receipt of myeloablative chemotherapy, use of alemtuzumab, delay in platelet engraftment, presence of acute graft-vs.-host disease (aGvHD), and underlying primary disease (7–9). Non-infectious neurologic sequalae were more common in patients with underlying metabolic or hematologic diseases whereas central nervous system (CNS) infections occurred more frequently in patients with an underlying immunodeficiency.

**Table 1:** Neurological complications following transplantation.

Hematopoietic Cell Transplantation	Solid Organ Transplantation
CNS infections	Cerebrovascular accidents
Encephalopathy/myelopathy of un- known causes	Drug-associated (calcineurin inhibitors, steroids) toxicity (including posterior reversible encephalopathy syndrome)
Cerebrovascular accidents	CNS infections
Irradiation/chemotherapy injury	CNS malignancy
Drug-associated (calcineurin inhibitors, steroids) toxicity (including posterior reversible encephalopathy syndrome)	
Metabolic encephalopathy	
Transplant-associated thrombotic microangiopathy	
CNS malignancy	
CNS graft-vs.-host disease (rare, diagnosis of exclusion)	

In the largest German adult allogenic transplant cohort of 2,628 patients who were followed for a median of 4 years, only 32 cases of viral encephalitis were reported; *Herpesviridae*, primarily human herpes virus-6 (HHV-6), was the most common causative agent (9, 10). In contrast, a recent cohort study from China of 30 haploidentical stem cell transplant recipients (median age of 25 years) with encephalitis, where HHV-6 incidence is reportedly lower than in the US, noted that only 13.4% were due to *Herpesviridae*. Unexpectedly, Respiratory Syncytial Virus (RSV) accounted for 50% of these cases with detection of the virus noted on polymerase chain reaction (PCR) analysis of the cerebrospinal fluid (CSF) (11–13). A recent review by Toomey et al. reported incidence of viral encephalitis by pathogen among HCT recipients and the highest incidence of 1.2% was due to RSV (10).

In pediatric solid organ transplant (SOT) recipients, rates and types of neurological complications are less well understood but include encephalopathy, cerebrovascular accidents, drug-associated toxicity, CNS malignancy, and CNS infections (Table 1) (14–17). Up to 30% of pediatric liver transplant recipients experience neurological complications compared to 50% of pediatric heart transplant recipients (16–19). Among heart transplant recipients, patients with a history of a ventricular-assistance device are at increased risk of cerebrovascular accidents (14). Pediatric liver transplant recipients with hyperbilirubinemia within the first seven days post-transplant carry a higher risk of calcineurin inhibitor-associated neurological complications (15). Rates of CNS infections vary by region and organ type and are not well reported in pediatric solid organ transplant recipients. In a Swiss cohort of 4,762 adult SOT recipients, 41 patients (0.86%) developed CNS infection, and 52.4% of these were due to viral etiologies. *Herpesviridae*, primarily HSV and VZV, remained the leading causative agents in this cohort, though none were secondary to HHV-6 (20).

While infectious encephalitides are not the most common etiology for neurologic complications in transplant recipients, it remains an important consideration in the evaluation of a transplant recipient who presents with fever along with altered mentation, new onset neurologic manifestations, or new onset or worsening headaches. Since viral etiologies predominate, using two clinical vignettes, this article will provide a contemporary review of meningoencephalitis secondary to *Herpesviridae*, polyomaviruses, adenovirus, arboviruses, donor-derived infections including lymphocytic choriomeningitis virus (LCMV), respiratory viruses, and enteroviruses in pediatric transplant recipients.

## Case 1

An 8 year-old male with past medical history of acute myeloid leukemia who underwent α/β-depleted T cell haploidentical bone marrow transplant (BMT) with engraftment on day +9, presented on day +21 with fever, rash, abdominal pain, vomiting, diarrhea, and irritability. The patient was without significant contact or epidemiologic exposures in the preceding 6 months prior to transplant, nor in his brief post-transplant period. Antimicrobial prophylaxis post-transplant included acyclovir (HSV and VZV recipient-positive), voriconazole and pentamidine. The BMT preparatory regimen included busulfan, cyclophosphamide, anti-thymocyte globulin (ATG), and rituximab. He did not have any known graft-vs.-host disease (GvHD) and was not receiving any post-transplant immunosuppression to prevent GvHD. Exam on initial encounter was notable for an irritable child, although appropriately oriented to person, place, and time, without meningismus or other neurologic deficits on exam. He had a diffuse macular, blanching, erythematous rash, sparing palms and soles. Labs were notable for evolving pancytopenia including lymphocytopenia. Blood cultures were drawn, and empiric antibiotics were initiated. A multiplex stool PCR panel was sent and returned negative. Quantitative polymerase chain reaction (PCR) from the blood for cytomegalovirus (CMV), Epstein-Barr virus (EBV), adenovirus were obtained and negative, however human herpes virus-6 (HHV-6) was detected in blood at 650,000 copies/ml. Evolving irritability, newly noted confusion, and delayed response to verbal cues prompted initiation of intravenous ganciclovir 5 mg/kg every 12 h, and lumbar puncture was pursued for CSF diagnostics. No CSF pleocytosis was observed, however HHV-6 was detected on the meningoencephalitis multiplex PCR panel in concert with a mildly increased CSF protein (65 mg/dl). CSF studies were negative for pathogens on multiplex PCR assay otherwise and there was no pleocytosis or hypoglycorrhachia. A brain MRI was subsequently performed and revealed non-specific, mild, symmetric increase in T2-weighted signal and restricted diffusion within the mid thalami bilaterally.

## Case 2

A 17 year-old male with history of complex congenital heart disease underwent heart transplantation. Induction consisted of 3 doses of ATG and methylprednisolone, followed by maintenance immunosuppression with tacrolimus, mycophenolate mofetil (MMF), and prednisone. Pre-transplant serostatus was notable for CMV donor negative/recipient negative, and EBV donor positive/recipient negative. Antimicrobial prophylaxis included acyclovir and trimethoprim/sulfamethoxazole. His course was complicated by early acute cellular rejection (ACR 1A/1R) and antibody mediated rejection (AMR 2) at 1 month post-transplant, prompting pulse methylprednisolone, and AMR management with initiation of rituximab, plasmapheresis, and bortezomib. Shortly after his AMR/ACR episode, he developed fever with worsening transaminases, followed by headache, confusion, and blurry vision in his right eye. Diagnostic workup was notable for new cytomegalovirus (CMV) detection in blood (7,678 IU/ml) and elevated liver transaminases (Alanine aminotransferase 230 IU/ml, Aspartate aminotransferase 194 IU/ml). A lumbar puncture was performed and was with detection of CMV by multiplex PCR in cerebrospinal fluid, a mild CSF pleocytosis (WBC 56 cells/mm^3^), and increase in protein (89 mg/dl). Multiplex PCR from the CSF was without other pathogen detection, as well as negative culture results from CSF and blood. Ophthalmologic examination revealed chorioretinal inflammatory/hemorrhagic changes in a perivascular pattern consistent with CMV retinitis within his right eye. MRI of his brain was obtained and noted mild leptomeningeal inflammation in addition to frontal lobe parenchymal T2 enhancement. The patient was started on intravenous ganciclovir at 5 mg/kg every 12 h.

## Clinical manifestations of viral meningoencephalitis in HCT and SOT

As described in cases 1 and 2, viral meningoencephalitis typically presents with fever and altered mentation. Venkatesan and colleagues proposed a more structured diagnostic criteria for meningoencephalitis or encephalopathy (Table 2). Neurological changes associated with meningoencephalitis also include impaired cognition, behavioral changes, speech disturbances, hemiparesis, and cranial neuropathies (21). Patients can also present with signs of nuchal rigidity if there is concomitant meningeal inflammation. Although the presence of symptoms is helpful, it is important to note that severely immunosuppressed patients may not have obvious signs of inflammation and thus, may exhibit subtle or atypical symptoms. As such, there should be a high index of suspicion for infectious etiologies in the differential diagnoses for transplant recipients who present with changes in mentation.

**Table 2:** Diagnostic criteria for meningoencephalitis (adapted from venkatesan et al.) (22).

**Major Criterion (required):**	Patients with altered mental status (altered level of consciousness, lethargy or personality change) lasting ≥24 h with no alternative cause identified
**Minor Criteria (2 required for possible meningoencephalitis; ≥3 required for probable or confirmed meningoencephalitis):**	Fever ≥38° C (100.4°F) within the 72 h of major criteria
	Newly noted generalized or partial seizures
	New onset of focal neurologic findings
	CSF WBC count ≥5/mm^3^^a^
	Acute or newly noted normality of brain parenchyma on neuroimaging suggestive of encephalitis
	Abnormality on electroencephalography that is consistent with encephalitis and not attributable to another cause

^a^ maybe absent in patients with neutropenia (ANC <500 cells/μl).

## Diagnostics

**Figure 1:**
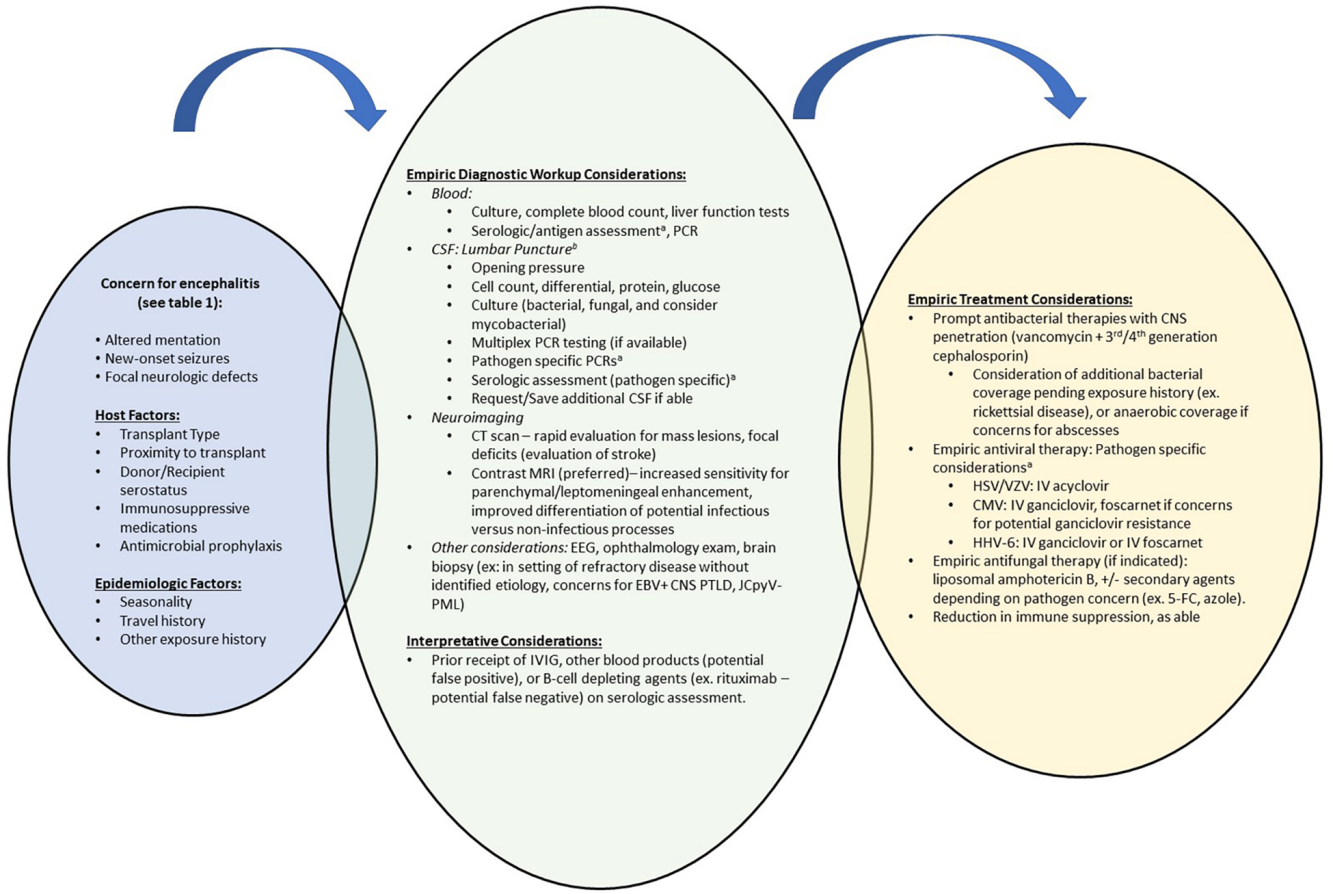
Suggested diagnostic and therapeutic approach to meningoencephalitis among HCT and SOT recipients. CMV, cytomegalovirus; CNS, central nervous system; CSF, cerebrospinal fluid; CT, computed tomography; EBV, Epstein-Barr Virus; EEG, electroencephalography; HCT, hematopoietic cell transplant; HHV-6, human herpesvirus-6; HSV, herpes simplex virus; IVIG, intravenous immunoglobulin; JCpvV, JC polyomavirus; MRI, magnetic resonance imaging; PCR, Polymerase chain reaction; PML, progressive multifocal leukoencephalopathy; PTLD, post-transplant lymphoproliferative disease; SOT, solid organ transplant; VZV, varicella zoster virus. ^a^See text for diagnostic recommendations, expected findings and empiric treatment recommendations for viral etiologies. Remainder of pathogen specific testing recommendations have been discussed by Venkatesan et al. (22). ^b^Based on diagnostic considerations, ensure adequate cerebrospinal fluid volume is requested for all anticipated tests.

Patients presenting with concerns of meningoencephalitis should undergo CNS imaging and lumbar puncture (Figure 1). In patients with viral meningoencephalitis, CSF is typically colorless and pleocytosis, if present, is <1,000 cells/mm^3^ with polymorphonuclear cells predominating initially, followed by lymphocytes thereafter (22). While Venkatesan's diagnostic criteria (Table 1) are largely applicable to transplant recipients, a lack of pleocytosis would not rule out meningeal inflammation or meningoencephalitis, especially in neutropenic patients. There may be a moderate increase in the CSF protein concentration but hypoglycorrhachia is uncommon. Multiplex PCR meningitis/encephalitis panels are a rapid and effective screening test for common pathogens including several viral etiologies. As the multiplex PCR panel does not have a sensitivity of 100% for any of the listed pathogens, dedicated viral PCRs should be obtained in those with high clinical suspicion. While viral culture and antigen-based testing for some viral etiologies are available, these are less commonly used and have been supplanted by molecular methods due to improved sensitivity and diagnostic turn-around time. Serum testing by serology or PCR may suggest an etiology, though detection of or absence from detection among serum specimens does not confirm nor disprove the diagnosis.

The diagnostic work-up for meningoencephalitis may not identify a causative etiology in 50% of cases (9, 11). However, identifying a pathogen helps determine appropriate anti-infective treatment options, inform prognosis, and guide need for preventative strategies to minimize recurrence. Metagenomic next generation sequencing on CSF samples appears promising, but its clinical utility is limited as results are typically not available for two weeks (23–27).

Neuroimaging findings are largely non-specific and similar areas of involvement may be shared by more than one pathogen. For example, temporal lobe involvement may be seen in many *Herpesviridae* meningoencephalitides whereas thalamic involvement is more commonly seen in arboviral CNS infections, but these findings are not exclusive to these pathogens (28, 29). When pursued, MRI with contrast is the imaging modality of choice, and particular attention should be paid to T2 weighted images, fluid attenuated inversion recovery (FLAIR) sequences, and diffusion weighted images (DWI) (22, 29–31). While DWI helps differentiate between cytotoxic and vasogenic edema and identify abnormalities that may be acute vs. remote, T2 weighted imaging and FLAIR sequences may detect early, subtle changes at the onset of an evolving infection or inflammatory process (32). Space-occupying lesions should prompt work-up for alternative etiologies such as abscesses or malignancies, whereas diffuse cerebral edema, infarction or hemorrhage may suggest a para-infectious processes.

Brain biopsy is infrequently performed but should be considered in cases where patients continue to deteriorate or, as part of the autopsy, in patients where neurological complications result in death. As with any other tissue sample, immunohistochemical staining and PCR analysis of the tissue should be pursued along with histopathology to evaluate for viral cytopathic effects.

Table 3 summarizes epidemiology, manifestations of neuroinvasive disease, diagnostics, treatment and outcomes of various viral pathogens discussed below.

**Table 3:** Summary of viral etiologies that cause infectious meningoencephalitis in pediatric transplant recipients.

Etiology		Epidemiology	Forms of CNS disease	Diagnostic testing and EEG findings, when available	Treatment	Outcomes	References
Adenovirus		HCT: • Incidence of adenovirus infection, 11–13% • Occurs prior to d + 100 • Risk factors: UCT, use of T-cell depleted allograft, CD3 + T < 300 cells/μl, receipt of ATG, and grade II-IV aGVHD SOT: • Incidence of adenovirus infection, up to 57% in intestinal, multivisceral transplant • Timing varies widely but occurs at median of 1 month post-transplant • Risk factors: type of SOT (intestinal is the highest risk), lymphocytopenia, use of T-cell directed immunosuppression (alemtuzumab, ATG), less than 5 years of age CNS disease is rare in both HCT and SOT	Fatal encephalomyeloradiculitis Acute limbic encephalitis	CSF or brain parenchymal tissue: molecular/PCR-based testing for adenovirus Histopathology: • Gold standard for tissue invasive disease • Involves immune-peroxidase and *in situ* hybridization staining that reveal cells with large nuclei with basophilic inclusions and a thin rim of cytoplasm also known as ’smudge cells’ EEG: epileptiform activity within involved areas, typically the frontotemporal lobes	Decrease IS Cidofovir and brincidofovir likely ineffective as they may not reliably penetrate CSF May consider adoptive immunotherapy but limited studies to comment on efficacy	CFR: 17% (HCT) to 63% (SOT with adenovirus hepatitis) Of two pediatric allogeneic HCT patients with adenovirus CNS disease, only one survived and the other developed chronic neurological deficits	(33–43)
Arboviruses		Pathogen transmission specific vectors (*Aedes, Colquillettidia, Culex* mosquitos, *Ixodes* tick). Donor derived infection known to occur.	Meningoencephalitis, Acute flaccid paralysis	Serologic testing of serum and CSF Cross-reactivity of serologic testing among arboviruses exist, plaque-reduction and neutralization testing needed for confirmatory diagnostic purposes PCR based diagnostics using CSF sample available for some arboviruses, however sensitivity is limited	Supportive management Reduction in IS IVIG has been employed with unclear efficacy	WNV–33% mortality rate among HCT and SOT recipients CFR remains largely unknown for other arboviruses given rarity of disease	(44–52)
*Herpesviridae*	CMV	HCT: • Incidence of 1–2% (early post-transplant) to 9% (1 year post-transplant) • CNS disease occurs in less than 1% and typically late post-transplant, median 178 days, range 80–285 days • Risk factors include CMV seropositivity mismatch (D-/R+), T-cell lymphopenia and GHD, underlying primary immunodeficiency or lymphoid malignancy	Meningoencephalitis Ventriculoencephalitis Myelitis CMV CNS disease should prompt evaluation for CMV retinitis	PCR-based detection of CSF (sensitivity ∼100%) and blood for CMV CMV meningoencephalitis: • Proven: CMV detection in brain parenchymal tissue • Probable: CMV detection on CSF CSF detection of CMV should be followed by resistance testing, using both the CSF sample and blood sample, if DNAemia is present. Molecular genotypic assays are preferred	Ganciclovir 5 mg/kg every 12 h IV If CNS involvement develops while receiving ganciclovir, patient should be switched to foscarnet 180 mg/kg/day divided every 8–12 h IV	Mortality rate was high: 75% (HCT) and 67% (SOT) Patients who survived CMV meningoencephalitis demonstrated improved immune reconstitution or received cellular or humoral adjunctive therapies, in addition to antiviral agents	(34, 53–69)
		SOT (prior to prophylaxis): • Incidence of 15% (kidney transplant) to 40% (liver transplant) • Incidence of CNS disease is very low but occurs within the first 3 months of transplant • Risk factors include CMV seropositivity mismatch (D+/R-), induction or augmented IS, lung and intestinal transplant recipients CMV meningoencephalitis most commonly seen in transplant recipients with clinically presumed or proven ganciclovir-resistant strains					
	EBV	EBV CNS disease (encephalitis and PTLD) post HCT and SOT is infrequent EBV + PTLD in HCT: • Incidence: 1–3.5% • Timing post-transplant: varies but up to 68% occurs in the first 100d post-HCT • Risk factors: T-cell depleting agents, graft manipulation, and donor EBV + serostatus EBV + PTLD in SOT: • Incidence: varies by transplant type, 1–20% • Timing post-transplant: generally within the first 2 years post-SOT • Risk factors: primary EBV infection, receipt of ATG, graft type, younger age, and intensity of immunosuppression	Encephalopathy Ataxia “Alice in Wonderland” syndrome Focal defects if CNS mass lesions (EBV + PTLD)	Serum PCR can detect viral load (high NPV for EBV + PTLD) CSF PCR may be obtained, though may be confounded by number of CSF lymphocytes PTLD requires tissue for diagnosis Serologic testing may have limited utility in ICH EEG: diffuse slowing consistent with meningoencephalopathy	Reduction in IS Pursue chemotherapeutic regimens for EBV + PTLD Intrathecal rituximab may be considered for CNS + PTLD EBV-specific VSTs are an emerging approach to care	Mortality rate with EBV + PTLD–20%	(70–75)
	HHV-6	HCT: • About 1.1%, up to 9.9% in umbilical cord blood transplant recipients • Risk factors include umbilical cord transplant, CD45RA– graft, underlying hematological malignancy or solid tumor, unrelated donor, and sex-mismatched donor • More common cause of infectious meningoencephalitis in the first 100 days post-transplant SOT: incidence, risk factors and time of onset is not well defined	Acute limbic encephalitis PRES SIADH HHV-6 meningoencephalitis	Serum and CSF testing for HHV-6 using a quantitative PCR assay see text about ciHHV-6	Reduce IS High-dose ganciclovir 5 mg/kg every 12 h IV or foscarnet 180 mg/kg/d divided every 8–12 h IV	In adult HCT with acute limbic encephalitis and imaging evidence of secondary amygdala or hippocampal atrophy, long term neurological deficits including expressive aphasia, intermittent mental status changes, and memory deficits have been reported	(9–11, 76–81)
	HSV	HCT (recipient +): 80% reactivation (in the absence of prophylaxis) before d + 365 SOT (recipient +): 9.8%	HSV meningoencephalitis	CSF PCR for HSV (sensitivity and specificity 95–100%), preferred, or viral culture (low sensitivity and longer turn-around time) If concerns for antiviral resistance arises, culture-based plaque-reduction assay is preferred EEG: periodic lateralizing epileptiform discharges	Reduce IS High dose acyclovir IV: • <3 months of age: 20 mg/kg every 8 h IV; up to 20 mg/kg every 6 h in neonates at 36–41 PMA based on a pharmacokinetic modelling study • 3 months–12 years of age: 15 mg/kg every 8 h IV • >12 years of age: 10 mg/kg every 8 h IV	Long-term outcomes of HSV meningoencephalitis are not well reported in post-infantile age groups	(9, 21, 34, 82–84)
	VZV	HCT: 0% (some older studies report up to 1.3%) SOT (recipient +): 11%%	VZV meningoencephalitis Ramsay-Hunt syndrome VZV myelitis Vasculopathy, VZV-associated stroke	CSF PCR for VZV VZV IgG from VZV EEG: periodic lateralizing epileptiform discharges	Reduce IS High dose acyclovir IV: • <3 months of age: 20 mg/kg every 8 h IV; up to 20 mg/kg every 6 h in neonates at 36–41 PMA based on a pharmacokinetic modelling study • 3 months–12 years of age: 15 mg/kg every 8 h IV • >12 years of age: 10–15 mg/kg every 8 h IV	None reported in transplant recipients but in VZV-unimmunized, immunocompetent children (*n* = 84) with CNS disease, 39 (23%) had neurological sequalae (seizure disorder or neurological deficits) and 3 (3.5%) died	(9, 10, 21, 22, 83–86)
Enteroviruses		Frequent infection among HCT and SOT recipients, however CNS involvement is rare and limited to case reports Humoral deficiencies increase risk for progressive CNS disease	Meningoencephalitis Seizures	PCR remains diagnostic testing modality of choice	Reduction in IS IVIG frequently employed in those with systemic disease due to enteroviruses	None available in transplant recipients Mortality 44% in patients with hypogammaglobulinemia Outcomes often favorable in those with non-severe (non-CNS) disease	(98, 99)
Polyomaviruses	BK polyomavirus	Renal transplant: 5–15% HCT: 8–25% Risk factors for BKPyV infection include immunosuppressed state, tacrolimus use, younger recipient age, ureteral stenting, ATG exposure, and obstructive uropathy CNS disease is rare	BKPyV-associated meningoencephalitis	PCR-based detection of CSF for BKPyV although false positives have been reported Diagnostic criteria for BKPyV meningoencephalitis: not well established but share similar imaging findings as PML	Decrease IS	Unknown	(34, 87–89)
	JC polyomavirus	Heart and lung transplant: 1.24 per 1,000 post-transplant patients years Rare in other SOT and HCT Risk factors for JCPyV-PML: CD4 + lymphocytopenia, older age (rare in patients less than 21 years of age), alloCT, receipt of two or more IS agents; occurs at median of 11 months (HCT) to 27 months (SOT) CNS disease is rare	JCPyV-associated PML	PCR-based detection of CSF for JCPyV although false negatives have been reported Diagnosis of JCPyV-PML requires either: • Histopathological criteria: ○ classic triad of demyelination, bizarre astrocytes and enlarged oligodendroglial nuclei, ○ CPyV detection via immunohistochemistry or electron microscopy, and/or ○ PCR-based detection of JCPyV OR • Clinical criteria: ○ Clinical features ○ PCR-based detection of JCPyV ○ Radiologic features: white matter demyelination and multifocal, hyperintense lesions of the subcortical and juxtacortical white matter that enlarge and coalesce over time	Timely decrease in IS while disease progression is limited Adoptive immunotherapy is an ongoing area of study; JCPyV-specific VST have shown better outcomes compared to BKPyV-specific VST for treatment of PML	High mortality: 40–100% (HCT), 84% (HCT and SOT) Median time between symptom onset and death: 19.5 months (HCT), 6.4 months (SOT)	(34, 90–96)
Respiratory viruses	Influenza, RSV, hMPV	Incidence: HCT—respiratory viral infection of 16.6% SOT—10% (Influenza), 11% (hMPV), 22% (RSV) Risk factors: steroid exposure, neutropenia, and lymphopenia are commonly reported but unclear if truly associated with viral infection Rates and risk factors for CNS disease are not known	Meningoencephalitis Acute necrotizing encephalopathy Myelitis Guillain-Barre syndrome Febrile seizures Cerebellitis	PCR-based detection of CSF and, if available, brain parenchymal tissue	Ribavarin and IVIG, although neither with clear benefit (RSV)	Mortality rate of 15% (RSV encephalitis) but none reported for influenza or hMPV	(11, 34, 97)

ATG, anti-thymocyte globulin; aGVHD, acute graft vs. host disease; BKPyV, BK polyomavirus; CFR, case fatality rate; ciHHV-6, chromosomally integrated human herpesvirus-6; CMV, cytomegalovirus; CNS, central nervous system; CSF, cerebrospinal fluid; CT, computed tomography; EBV, Epstein-Barr Virus; EEG, electroencephalography; GVHD, graft vs. host disease; HCT, hematopoietic cell transplant; HHV-6, human herpesvirus-6; hMPV, human metapneumovirus; HSV, herpes simplex virus; ICH, immunocompromised host; IgG, immunoglobulin G; IS, immunosuppression; IV, intravenous; IVIG, intravenous immunoglobulin; JCpvV, JC polyomavirus; MRI, magnetic resonance imaging; NPV, negative predictive value; PCR, Polymerase chain reaction; PMA, post-menstrual age; PML, progressive multifocal leukoencephalopathy; PRES, posterior reversible encephalopathy syndrome; PTLD, post-transplant lymphoproliferative disease; RSV, respiratory syncytial virus; SIADH, syndrome of inappropriate antidiuretic hormone; SOT, solid organ transplant; UCT, umbilical cord transplant; VST, viral-specific T cells, VZV, varicella zoster virus; WNV, west nile virus.

## Causative viral pathogens

### HHV-6

HHV-6, a Betaherpesvirinae, is neurotropic and may present with CNS disease in immunocompromised patients. While the replicating virus is often shed in saliva, the latent form can be found in mononuclear cells. Most cases of HHV-6 infection in transplant recipients are presumed to occur because of reactivation of latent virus rather than newly acquired infection through community transmission (21, 33, 34, 100, 101).

HHV-6 reactivation in allogeneic HCT recipients may present with fever, rash, bone marrow suppression, pneumonitis, GVHD, and graft rejection (34). When it causes meningoencephalitis, patients present with confusion, seizures and may evolve to have anterograde or retrograde amnesia. HHV-6 meningoencephalitis may also present with choreoathetosis or mimic posterior reversible encephalopathy syndrome (76, 102). Some patients also develop syndrome of inappropriate antidiuretic hormone secretion, and although not unique to HHV-6, sodium disorders are more commonly reported in HHV-6 meningoencephalitis compared to other infectious encephalitides (10, 33). At an incidence of 1.1%, HHV-6 is the most common cause of known viral meningoencephalitis reported in HCT recipients (10).

Among adult SOT recipients, reactivation leading to HHV-6 viremia has been reported in up to 40% of patients, however only about 1% develop HHV-6 disease and none develop neurologic sequalae (103, 104). In an adult Swiss cohort of 4,762 adult SOT recipients, HHV-6 was not reported as a causative agent of viral meningoencephalitis among 42 cases of infectious meningoencephalitis (20). While HHV-6 meningoencephalitis is uncommon in SOT recipients, characteristic manifestations of HHV-6 disease include myelosuppression, interstitial pneumonitis, hepatitis, colitis (105). Asymptomatic HHV-6 viremia has also been reported and typically does not warrant treatment.

The diagnostic approach to evaluate for HHV-6 meningoencephalitis should include both serum and CSF testing for HHV-6 using a quantitative PCR assay. HHV-6 DNAemia of 10,000 copies/ml or more has been reported to be strongly associated with HHV-6 end organ disease, however a viral load threshold that warrants antiviral therapy is not well established and depends largely on evidence of clinical disease (77). Additionally, HHV-6 detection by PCR does not imply disease as it may reflect detection of HHV-6 genome integrated into the telomere regions of the host chromosome, also known as chromosomally integrated HHV-6 infection (ciHHV-6).

About 1% of the immunocompetent population and 1%–3% of transplant recipients carry ciHHV-6 (106). ciHHV-6 can be readily diagnosed when the HHV-6 level in whole blood is 1:1 with the human genome, such that the viral load is greater than 10^6^ copies/ml when assuming that there are 4–10 million leukocytes/ml of blood (107). ciHHV-6 may formally be differentiated from replicating HHV-6 viral load in the blood using molecular cytogenetic analysis or droplet digital PCR (108). A matched cohort study by Heldman and colleagues comparing allogeneic HCT with ciHVV-6-positive donor and recipients to allogeneic HCT with ciHHV-6-negative donor and recipients noted no differences in CNS symptoms between both groups. However, this study was limited by a low incidence of HHV-6 meningoencephalitis (3%), and only one case each of clinically determined HHV-6 meningoencephalitis in the ciHVV-6-positive and ciHVV-6-negative groups (109).

When HHV-6 is detected using PCR for samples other than blood, such as CSF or tissue, it should be interpreted in the appropriate clinical context. Given that normal CSF may contain up to 5 nucleated cells/μl, ciHHV-6 may be detected on the multiplex PCR meningitis/encephalitis panel with minimal leukocytes (106). In contrast, if HHV-6 is noted in the background of gross viral cytopathic effects on a tissue sample and other etiologies have been ruled out, then this likely reflects pathogenic HHV-6 infection rather than chromosomal integration.

In any transplant recipient presenting with acute signs of encephalopathy and concomitant HHV-6 detection at any viral load above the reference threshold in the CSF, HHV-6 meningoencephalitis should be assumed, and antiviral treatment should be initiated while a broad work-up for other etiologies is being pursued. While a higher all-cause mortality rate was seen in patients being treated with ganciclovir compared to foscarnet, this difference is not significant when adjusted for other confounders (110). Studies comparing monotherapy to combination therapy with both foscarnet, and ganciclovir have not elucidated any benefit in outcomes of HHV-6 meningoencephalitis (111–113). Duration of therapy is not well established but expert consensus recommends at least three weeks of therapy followed by retesting of the CSF to ensure viral clearance has been achieved (112, 113).

While uncommon, persistent or increasing HHV-6 DNAemia in the setting of worsening neurological function despite ongoing antiviral therapy in a transplant recipient should raise suspicion for antiviral resistance however resistance testing is only available in a research setting. Mutations in the U69 and U28 HHV-6 genes confer ganciclovir resistance and would warrant a switch to intravenous foscarnet or consideration of adoptive immunotherapy (114, 115). Adoptive immunotherapy using third-party or donor virus-specific T lymphocytes (VST) has been used successfully in one patient presenting with HHV-6 meningoencephalitis, however larger efficacy studies are needed to recommend routine use in this scenario (116).

Cidofovir and brincidofovir retain some activity against HHV-6 but they have the lowest EC_50_ values *in vitro* and, consequently, are less preferred antiviral agents. Additionally, Cidofovir does not penetrate the blood-brain barrier and would not be an appropriate option in cases of HHV-6 meningoencephalitis. Brincidofovir is currently not commercially available (117).

Developmental and long-term neurological outcomes of pediatric transplant recipients with HHV-6 meningoencephalitis are not well studied. In an adult, Japanese, HCT cohort, higher levels of IL-6 in the CSF were associated with increased risk of mortality in patients with HHV-6B meningoencephalitis (118).

### HSV

Up to 27% of late adolescents have been exposed to HSV-1 and up to 21% have been exposed to HSV-2 in the US (82). In addition to reactivation from its latent state in neuronal cells, HSV infection may be acquired via community transmission through exposure to mucosal secretions where it can be intermittently shed in asymptomatic, immunocompetent individuals. Donor derived HSV infection, albeit rare, has been reported in a liver transplant recipient who developed hepatitis and meningoencephalitis (119).

Clinical manifestations of HSV disease in transplant recipients include mucocutaneous vesicles, hepatitis, infection of the adrenal glands, ocular disease (keratitis, endotheliitis, uveitis, retinitis), pneumonitis, and meningoencephalitis. HSV CNS disease may present as ataxia, cranial nerve palsies including anosmia, temporal lobe seizures (apraxia, lip smacking), olfactory hallucinations, behavioral abnormalities, and psychiatric changes (101). While HSV results in sporadic cases of fatal meningoencephalitis in immunocompetent patients, institution of prophylactic measures against HSV make these viral etiologies less likely to cause meningoencephalitis in transplant recipients (120).

In a transplant recipient presenting with vesicular rash and altered mentation, HSV or VZV meningoencephalitis should be strongly suspected, and empiric treatment should be initiated given a possible clinical diagnosis. If a lumbar puncture cannot be pursued in the immediate period, swab of an unroofed, mucocutaneous vesicle should be sent for molecular analysis to evaluate for HSV and VZV.

Cerebrospinal fluid may be without pleocytosis in the absence of meningeal inflammation, however CSF erythrocytosis may be present (85). Confirmed HSV meningoencephalitis requires detection of HSV from CSF or brain tissue specimens, typically by PCR. Importantly, up to 10% of patients with HSV meningoencephalitis may not have detectable HSV by PCR of CSF in the first two days of symptom presentation. Thus, a negative CSF PCR should be a followed up with a repeat lumbar puncture 3–7 days later for repeat CSF molecular analysis should high clinical suspicion remain (101). Additionally, in patients without humoral immunodeficiency or muted antibody responses, CSF HSV IgM, in the initial 2–3 weeks of infection, and IgG may be sent if sufficient CSF sample is available (22, 86).

Viral culture of CSF is typically reserved for cases where concerns for antiviral resistance arise. Mutations in the thymidine kinase or DNA polymerase genes confer resistance to acyclovir. Phenotypic resistance testing, the current gold standard, utilizes a culture-based plaque-reduction assay (82). Antiviral resistance testing may be of particular importance in patients who develop HSV breakthrough disease, despite being on antiviral prophylaxis, or patients who continue to neurologically deteriorate or develop worsening disseminated disease on high dose intravenous acyclovir (34).

Once diagnosis has been established, treatment with high dose intravenous acyclovir should be continued for a minimum of 21 days. A repeat lumbar puncture should be performed prior to cessation of therapy to ensure viral clearance. If viral detection persists in the CSF, therapy should be continued, and a follow-up lumbar puncture may be pursued weekly to evaluate for viral clearance. As with most other viral encephalitides in transplant recipients, reduction in immunosuppression should be strongly considered if feasible.

In patients with acyclovir-resistant strains, either due to clinical failure despite ongoing high dose acyclovir adjusted for expected body weight while awaiting resistance testing or confirmed antiviral resistance to acyclovir, intravenous foscarnet 90 mg/kg every 12 h is an appropriate alternative (121).

Secondary prophylaxis or chronic suppressive therapy is not routinely recommended in transplant recipients with HSV or VZV meningoencephalitis. However, it may be considered on a case-by-case basis, especially in patients who developed viral disease in the setting of augmented immunosuppression, history of recurrent HSV (2 or more episodes in the prior year), or have evidence of profound lymphocytopenia (CD3 + T < 300 cells/μl).

### VZV

VZV seroprevalence by late adolescence is 93.6% and likely reflects seroprotection post-immunization (122). VZV becomes latent in neuronal cells and may reactivate in immunosuppressed states to cause disease, although this is less commonly seen with the Oka or VZV vaccine strain.

In patients with VZV, clinical manifestations of disease are similar to HSV, and cutaneous findings may occur later than other end-organ involvement including meningoencephalitis (123). However, neurological complications in VZV may include Ramsay-Hunt syndrome, myelitis and vasculopathy, or VZV-associated stroke (85). Cases of fatal VZV meningoencephalitis are rare among transplant recipients given the wide institution of prophylactic measures against HSV and VZV in seropositive patients in the early post-transplant period when the risk of viral reactivation is higher (120, 124, 125).

As in the case of HSV, in a transplant recipient presenting with vesicular rash and altered mentation, HSV or VZV meningoencephalitis should be strongly suspected, and empiric treatment should be initiated given a possible clinical diagnosis. The swab of an unroofed, mucocutaneous vesicle should be sent for molecular analysis to evaluate for HSV and VZV if a CSF sample cannot be readily obtained for testing.

When a lumbar puncture is pursued, CSF sample should be sent for both molecular analysis and VZV IgG as CSF IgG is more sensitive than the CSF PCR assay in cases of VZV cerebral vasculopathy (22, 86). In a patient with inadequate antibody production or muted antibody responses, the lack of CSF seropositivity should be interpreted with caution.

Viral culture of CSF is typically reserved for cases where concerns for antiviral resistance arise and a similar approach to HSV testing may be employed with preference for the culture-based plaque-reduction assay; this is not available commercially (82). However, acyclovir-resistance is much less common in VZV disease (34).

On brain MRI, VZV meningoencephalitis may be accompanied by ischemic infarcts, vasculopathy and encephalomalacia (34, 123).

In patients receiving treatment for VZV CNS disease, high dose intravenous acyclovir should be continued for a minimum of 21 days. If viral detection persists in the CSF prior to the cessation of therapy, acyclovir should be continued, and a follow-up lumbar puncture may be pursued at one week intervals until viral clearance has been established. Reduction in immunosuppression should also be strongly considered if feasible. Secondary prophylaxis is not standard of care but may be considered in certain circumstances as outlined in the HSV section.

### CMV

CMV seroprevalence in the US ranges from 28% in children less than 5 years of age, 25%–80% in adult women to 48% in adult men (126, 127). As a Betaherpesvirinae, it establishes latency in human cells (stem cells in bone marrow, epithelial cells of the kidney and salivary glands) with the potential to reactivate (101). Infection in transplant recipients may occur due to endogenous reactivation (recipient or donor-derived) or exogenous exposure to mucosal secretions of infected individuals.

CMV meningoencephalitis is thought to occur secondary to disseminated viral invasion of host cells following which CMV-infected macrophages or monocytes translocate to the CNS or through direct viral invasion of the CSF (53). Since this process is subacute, active viremia may not be present at the time of diagnosis of CMV meningoencephalitis, and thus absence of viremia should not exclude CMV CNS disease (128).

Clinical symptoms in patients with CMV meningoencephalitis include progressive neuropsychological dysfunction, such as impaired memory and inability to concentrate, and subacute onset of motor or sensory deficits, cranial nerve palsies, ataxia, and hemianopia. Ventriculoencephalitis presents with more rapidly evolving symptoms and has been described in allogeneic HCT patients with GvHD (53–55).

Among 17 allogeneic HCT patients (six pediatric) with CMV meningoencephalitis, five (29%) patients also developed CMV retinitis. Most cases (94%) of CMV meningoencephalitis were preceded by recurrent ganciclovir-resistant CMV DNAemia (82%). The remainder had refractory CMV DNAemia while receiving ganciclovir, however 6 patients (35%) did not have active CMV DNAemia at the time of CMV meningoencephalitis (54–59). Of the four patients who survived (three pediatric), two demonstrated a robust cellular immune recovery and another had robust humoral immune recovery. Therapeutically, one received adjunctive intravenous CMV-hyperimmune globulin, two received pooled immunoglobulin, intrathecal CMV- hyperimmune globulin, and intravenous CMV-directed VST (54, 56, 58).

Among two reported cases of CMV encephalitis in adult SOT recipients and one pediatric case reviewed here, one patient had active, ganciclovir resistant, CMV DNAemia at the time of diagnosis of CMV meningoencephalitis (60, 61).

When considering diagnosis of CMV meningoencephalitis, the CSF multiplex PCR panel provides qualitative screening for CMV but obtaining a quantitative PCR value on the CSF is encouraged to allow providers to trend viral load in response to therapies. CSF viral culture has poor sensitivity (18%) and thus may not be a reliable diagnostic modality (53). On brain tissue specimens, aside from large intranuclear or intracytoplasmic inclusions, histopathology may reveal necrosis at sites of viral invasion in addition to viral cytopathic effects (53). All patients with probable CMV meningoencephalitis or suspected CMV meningoencephalitis with ongoing DNAemia should be evaluated for CMV retinitis (56, 129).

A majority of the cases of CMV meningoencephalitis have been reported in patients with clinically presumed or proven ganciclovir-resistant strains but some have noted mutant strains in the CSF and wild type stains in the peripheral blood (54–59). As such, a CSF sample with confirmed molecular detection of CMV should be sent for resistance testing (commercial testing is available currently through University of Washington), separate from peripheral blood. Antiviral resistance should be suspected in patients who have less than 1 log reduction in DNAemia despite two weeks of appropriately dosed antiviral therapy, also known as refractory infection (68, 130). Additionally, CMV resistance testing should also be pursued from a CSF sample in patients who develop CMV meningoencephalitis in the absence of active DNAemia. Culture-based phenotypic assays and molecular genotypic assays (preferred) are available for antiviral resistance testing targeting UL 54 DNA polymerase, UL 56 terminase complex and UL 97 kinase (62, 63, 68). However, false negatives on these assays remain a concern (130).

In patients with UL97 mutation conferring resistance to ganciclovir, foscarnet should be instituted. In patients with UL54 mutation conferring resistance to foscarnet, alternative therapies with effective CNS penetration are limited (130). Maribavir does not cross the blood brain barrier and cannot be used for CNS disease as it has not been studied in these cases. While there are case reports utilizing letermovir for CMV retinitis, it is not recommended as monotherapy for active CMV disease. Similarly, cidofovir is not an appropriate alternative due to a paucity of data on CNS penetration and, efficacy (130).

Reduction in immunosuppression, as feasible, to optimize cellular immune response and enhance clearance of CMV is recommended. Mammalian targets of rapamycin inhibitors, such as sirolimus and mTOR inhibitors, are associated with a lower risk for CMV infection and may be an appropriate substitute to other immunosuppressants, particularly as maintenance immunosuppression in SOT recipients with CMV meningoencephalitis.

Adoptive immunotherapy is a promising therapeutic consideration and may be the only option in cases of UL54 and UL97 mutant strains, although efficacy studies are lacking (54, 131). Two pediatric HCT recipients who received CMV-specific VST showed complete recovery after CMV meningoencephalitis in this otherwise fatal disease (54).

### EBV encephalitis and central nervous system post-transplant lymphoproliferative disease (CNS + PTLD)

Epstein Barr Virus (EBV), a Gammaherpesvirine, is a frequently encountered viral infection, with a lifetime seroprevalence of 80%–90% (132). EBV establishes latency predominantly among circulating B-cells, though integration within T and NK-cells are possible. Among HCT and SOT recipients, donor seropositivity for EBV represents the largest risk factor for development of EBV infection, though children are uniquely predisposed to de-novo infection given the lower likelihood of infection with EBV prior to transplant. EBV DNAemia post HCT is present in roughly 28%–54% of recipients, with likelihood of detection affected by the presence of T-cell depleting agents, graft manipulation, and donor EBV + serostatus. In a study of adult HCT recipients with and without T-cell depletion, the median time to EBV detection in blood was 58 and 63 days, respectively (133). EBV PTLD was observed in 12% of the study cohort, however only in those with T-cell depleted grafts. Among SOT recipients, general risk factors for PTLD development include primary EBV infection, receipt of ATG, graft type, younger age, and intensity of immunosuppression (71). Chronic, high viral load EBV DNA detection in the blood of pediatric heart transplant recipients is proposed to increase the risk of subsequent PTLD development (134), however this has not been capitulated among other pediatric SOT groups (135, 136).

Typical symptoms among immunocompetent individuals with EBV include clinically asymptomatic infection to infectious mononucleosis-like symptoms (132). EBV meningoencephalitis among immunocompetent patients may present with altered mentation and an “Alice in Wonderland” syndrome. While rare, meningoencephalitis and EBV + CNS PTLD represent serious complications of EBV infection post-HCT and SOT (70, 137). Focal neurologic defects may be present in the setting of EBV + CNS PTLD, depending on the size and location of the proliferative lesion.

Serologic assessment by EBV viral capsid antigen (IgG and IgM), as well as EBV nuclear antigen (EBNA IgG) is a common tool utilized in diagnosis of primary infection among immunocompetent children. However, in those with immune compromise, receipt of immunoglobulin or other blood products, or known history of EBV infection, serologic interpretation may be challenging. Use of EBV PCR from serum specimens can help identify reactivation of EBV in those with history of prior infection. EBV PCR of blood has a high negative predictive value for EBV + PTLD among SOT recipients, though the positive predictive value remains poor (71). EBV PCR from CSF may help improve diagnostic accuracy of EBV CNS mediated disease, though lymphocyte integration for EBV may result in false positive testing results and should be considered in the setting of significant CSF pleocytosis. Diagnosis of PTLD requires tissue confirmation of lymphoproliferation—the morphology of the lymphoproliferative lesion (e.g., early lesions, polymorphic, monomorphic PTLD) on histopathologic evaluation is necessary in determining the appropriate therapeutic approach (138). Thus, in patients with suspected EBV + PTLD, a tissue-based approach should be sought prior to pre-emptive management.

Reduction in immunosuppression, as able, is the mainstay of treatment of EBV DNAemia in HCT and SOT recipients (138, 139). Antiviral therapies, including intravenous ganciclovir, have been employed, though antiviral efficacy remains largely unknown, especially in the setting of viral reactivation of EBV (138). In those with EBV DNAemia in absence of lymphoproliferation, rituximab has been employed as a pre-emptive approach (139, 140); however, the data regarding its use in this remains relatively sparce, and thus routine use is not currently recommended (141). Chemotherapeutic interventions (cyclophosphamide, prednisone, rituximab) are utilized depending on the morphology of the PTLD lesion (140). EBV + CNS PTLD creates further challenges in therapy given the blood-brain barrier, and intrathecal rituximab has been utilized in management (141). Adoptive immunotherapy utilizing EBV viral specific T-cells (VSTs) have been employed in refractory cases of PTLD, though clinical trials to demonstrate efficacy are still needed (144).

Prior to 2000, mortality rates due to EBV + PTLD approached 85% post HCT (145). However, with introduction of monitoring and early interventions (ex. reduction in immunosuppression), morality rates have decreased markedly, though still remain substantial (146). A single center study on outcomes of pediatric EBV + PTLD yielded a mortality rate of 20% (72). CNS + PTLD disease is a risk factor itself for poor outcome (139).

### BK and JC polyomavirus

The polyomaviruses, BK and JC virus, cause human infection, though symptomatic disease rarely occurs outside the immunocompromised host. BK polyomavirus (BKPyV) exposure occurs earlier in life with seroprevalence rates reaching 90% by 4 years of age, whereas to JC polyomavirus (JCPyV) has a seroprevalence of 35% by adolescence (34). De-novo infection occurs through direct contact with mucosal secretions from previously infected individuals, due to lifetime viral shedding, or in renal transplant recipients, endogenously from the renal allograft (34, 90). Reactivation can occur from latent virus in the CNS (87). Polyomavirus meningoencephalitis is rare. Among transplant recipients, the most commonly known entity is JCPyV-associated progressive multifocal leukoencephalopathy (PML) (34, 88). Rare cases of BKPyV meningoencephalitis have been reported in HCT, renal and heart transplant recipients, including pediatric patients. Symptomatology is similar to that seen in JCPyV-PML (87, 147, 148).

Due to the progressive, subacute nature of presentation of JCPyV-PML, the median time to diagnosis from symptom onset was 1.6 months in a cohort of HCT and SOT recipients (91). The presence of JCPyV in the CSF correlates with the diagnosis (sensitivity of 72%–92%) of PML, however, false negative results have been reported and thus the lack of viral detection on CSF should not exclude the diagnosis (90, 92). PCR-based analysis of the CSF is also available for BKPyV although detection has also been reported in asymptomatic patients (87).

Diagnosis of JCPyV-PML, as proposed by the American Academy of Neurology, requires meeting either histopathological or clinical criteria. Clinical criteria include clinical features, radiologic findings (Table 3), and PCR-based detection of JCPyV on CSF (92). As a rapidly progressive demyelinating disorder of the CNS, clinical symptoms of PML depend on the areas of the pathological white matter involvement. This includes weakness, cranial nerve deficits (visual loss, ocular palsy, dysarthria), dysphagia, cognitive dysfunction including poor memory recall, motor deficits, sensory deficits, cerebellar symptoms including gait disturbance, personality or behavioral change, aphasia, and seizures (91, 93, 149). The absence of JCPyV detection on CSF in a patient with compatible clinical and radiological findings yields a possible clinical diagnosis. If JCPyV is detected but the patient only presents with either imaging or clinical findings, a probable clinical diagnosis of JCPyV-PML may be made. Definitive clinical diagnosis requires all three features of clinical criteria to be met (92). Independent of clinical criteria, definitive diagnosis using histopathology of brain tissue requires presence of at least two of three findings (Table 3). If not, a probable histopathologic diagnosis can be made on the presence of the classic triad alone and this can be revised to a definitive diagnosis in a patient with a possible clinical diagnosis. A possible histopathologic diagnosis requires detection of JCPyV on immunohistochemistry or electron microscopy evaluation of brain tissue (92).

Timely reduction in immunosuppression, ideally in the setting of limited disease progression, may improve likelihood of survival (90, 148, 150). Antiviral therapies trailed for JCPyV-PML have not prolonged survival or improved neurological outcome. Adoptive immunotherapy with PyV-VST is an ongoing area of study and provides a theoretical solution in the form of immune reconstitution. As BKPyV shares sequence homology to JCPyV in their immunodominant antigens, pilot studies using BKPyV-specific VST for treatment of PML have been initiated, though thus far yielding poor results (151, 152). In one study of the four HCT patients with JCPyV-PML who received BKPyV-specific VST, 75% died (152). However, T cells generated by stimulation against JCPyV antigens have shown more promise. In a mixed cohort of nine patients, within which two patients had undergone alloHCT and 3 patients had undergone autologous transplant, six (66.6%) had evidence of clinical improvement, radiological improvement, or clearance of JC virus on CSF after JCPyV-specific VST (94).

### Adenovirus

Serotype C is the most common adenovirus identified in humans (153) and while the extent of clinical disease varies greatly by the tissue tropism conferred to each serotype, cases of meningoencephalitis have been attributed to a variety of serotypes including species A (serotype 31), B (serotypes 3 and 7), C (serotype 2), and D (serotypes 26 and 49 mixed with 31) (154–157). Adenovirus is typically acquired from the community through intermittent shedding of the virus in the airways and stool. It is known to remain latent in lymphoid tissue and may reactivate. Among SOT recipients, adenovirus may be acquired from the donor allograft after a recent infection involving the allograft (lung, liver, kidney) or while actively viremic at the time of allograft extraction (34).

Adenovirus CNS disease is rare among HCT and SOT recipients but may be considered in patients with adenovirus disseminated disease. Among SOT, the increased propensity for viral infections among heart, lung, and intestinal transplant recipients are likely reflective of a higher net state of immunosuppression in comparison to liver or kidney transplant recipients (34).

When adenovirus meningoencephalitis is a concern in a transplant recipient presenting with altered mentation, evaluation for disseminated adenovirus disease should be performed. Isolated adenovirus disease in HCT recipients has been reported but is rare (35). In cases of meningoencephalitis, molecular re-evaluation of the CSF in response to therapy is not routinely recommended.

Features of adenovirus CNS disease on brain MRI in immunocompromised patients, including one patient with HIV and two pediatric patients who underwent allogeneic HCT, noted infiltrating hyperintensities at the fornix and chiasmatic structures which are otherwise not reported with other viral etiologies but nonetheless, non-specific to adenovirus infection (35).

When considering treatment, cidofovir, an analog of cytosine, inhibits adenoviral replication *in vitro* by inhibition of viral DNA polymerase (158). Data regarding use of cidofovir for CNS disease is scarce and inconclusive, and cidofovir levels were undetectable in the CSF of one patient with detectable serum levels (159). As such, alternative therapies should be pursued in cases of adenovirus CNS disease.

Brincidofovir, a lipid conjugate derivative of cidofovir, has better bioavailability and accumulates at higher concentrations than cidofovir intracellularly thereby exhibiting improved antiviral EC_50_ compared to cidofovir (34). However, there are no reports of its use in cases of CNS disease and none regarding penetration through the blood-brain barrier. Nonetheless, it is a safer and more potent alternative to cidofovir. Brincidofovir is currently not commercially available.

Adoptive immunotherapy using VST is a promising alternative, and while there are no cases of its use in adenoviral CNS disease, there has been a report of successful VST use in a patient with other virally mediated CNS disease suggesting possible antiviral activity in the CNS (54, 160, 161). Outcomes of adenovirus CNS disease reported in literature remain poor (35).

### Arboviruses

While infrequent, infectious meningoencephalitis by mosquito or tick (arthropod) borne vectors remain an important contributor to meningoencephalitis post-HCT or SOT given the associated morbidity. In general, a thorough epidemiologic history is needed among immunocompromised patients to better identify risk factors for arboviral meningoencephalitis. Pertinent arboviruses among HCT and SOT recipients are discussed below.

West Nile Virus (WNV), a *Flavivirus*, is the most common mosquito-borne viral disease in the United States (US), predominantly affecting older individuals. A recent single center study noted it as the most common viral detection among children with meningoencephalitis apart from *Herpesviridae* infections (44). WNV has been implicated to cause both donor derived infection as well as acquired neuroinvasive disease post-transplant with significant associated morbidity and mortality (45, 46). Data on WNV in pediatric HCT and SOT recipients is limited to case reports (47–49). Transmitted by the *Culex* mosquito, clinical presentation often develops between 3 and 14 days after inoculation with fevers and headache. Given the immunocompromised status of the host, progression to altered mentation, seizures, and coma may develop. Diagnosis is often made through serologic assessment of serum or CSF, though reverse-transcriptase polymerase chain reaction (RT-PCR) may improve diagnostic yield if performed early in the clinical presentation. Treatment is supportive (50); use of IVIG and interferon (IFN) alpha 2b has been reported in case series, though without clear evidence of efficacy (46). Additionally, given concerns of IFN potentially contributing to graft rejection, use of IFN-alpha 2b among SOT recipients has been limited (50). The overall case fatality rate is approximately 10% amongst individuals with WNV neuroinvasive disease. Mortality among pediatric HCT and SOT recipients has been reported in literature, comprising approximately 33% of documented cases (49, 51). This rate is comparable to adult studies, with a contemporary adult cohort noting mortality rate of 36% amongst SOT recipients with neuroinvasive WNV disease (46). Importantly, donor tissue screening for WNV is not mandatory, and varies regionally by the organ procurement organization. As regions of WNV endemicity in the US expand, targeted deceased donor screening, particularly in areas of high WNV infection burden, may be beneficial given the associated morbidity and mortality of WNV disease in SOT recipients (52).

St. Louis encephalitis virus (SLEV) is a *Flavivirus* first discovered in 1933, transmitted by the *Culex* mosquito. While most have clinically inapparent infection (99%), those that do develop symptoms will often present with signs and symptoms of encephalitis. SLEV may also be transmitted through blood products (162). Diagnosis is made through serologic or PCR detection, though serology may cross-react with other flaviviruses such as WNV or La Crosse Virus. Management is supportive. IVIG and interferon alpha 2b have similarly been used in case reports, though without clear evidence of efficacy (163). Mortality ranges from 5%-20% amongst those with SLEV encephalitis, though outcome data amongst HCT and SOT recipients is limited.

Eastern equine encephalitis virus (EEEV) is an *Alphavirus* with transmission to humans through the *Colquillettidia*, *Culex* and *Aedes* mosquito species. Cases are infrequent in the US, with roughly 8 cases reported per year (CDC ArboNET). Pediatric data remains limited—a case series from 2013 described 15 total cases collected from 1970 to 2010, with the most common clinical presentations including fever, headache, and seizures (164). Donor derived infection among SOT recipients is reported, with severe disease noted on acquisition (165). Diagnosis is made primarily through serologic assessment. Treatment is supportive, though IVIG has been employed without clear evidence of success (165). Mortality rates approach 30% in those affected, typically among older adults. Severe neurologic deficits (33%) and death (27%) predominated outcomes among the pediatric population with EEEV encephalitis (164), however mortality specific to pediatric HCT and SOT recipients is unknown.

Powassan virus is a *Flavivirus* transmitted by the *Ixodes* tick. Few cases have been reported in the US (189 cases total 2012-2021, CDC ArboNET), with most symptomatic individuals presenting with fever, headache, and weakness (166). Among children, cases remain limited predominantly to case reports (167). No cases of Powassan virus infection among pediatric SOT recipients have been reported, though an adult renal transplant recipient developed acute Powassan virus meningoencephalitis with transmission through receipt of blood products (168). Diagnosis is made primarily through serologic evaluation. Treatment is largely supportive, with IVIG utilized without clear evidence of efficacy (166). Mortality is reported between 10% and 15% of affected cases in the general population.

Among these important considerations, one notable absence is La Crosse Virus (LACV). LACV is the most common neuroinvasive arboviral infection among children in the US (169). While most affected individuals are asymptomatic, those who develop symptomatology are often younger in age (pediatric) and will present with symptoms concerning for meningoencephalitis. Nevertheless, among the 3 largest case series inclusive of >300 pediatric cases, no patient had an underlying immunocompromised status (169–171).

### Enteroviruses

Enteroviral meningoencephalitis is the most common cause of viral CNS disease among children, predominantly affecting newborn and infant children (44). Classic presentations of enteroviral meningoencephalitis among children include fevers, headache, altered mental status, as well as seizure like activity or focal neurologic deficits. CSF analyses of immunocompetent patients reveal a polymorphonuclear (neutrophilic) predominance. Literature regarding enteroviral disease among immunocompromised children comprises primarily of disease in those with impaired humoral immune responses, particularly X-linked agammaglobulinemia. Chronic enteroviral meningoencephalitis has been described in this population, with a high rate of associated morbidity and mortality (172). Clinical presentation includes altered mentation, weakness, and seizure activity among affected individuals. Chemotherapeutic regimens targeting B-cell populations (e.g., rituximab), and resultant hypogammaglobulinemia have produced similar disease presentations, with a mortality rate approaching 44% of affected individuals (98).

Despite the frequency of enteroviral infection, enteroviral meningoencephalitis post-transplant remains infrequently reported in literature. A recent study evaluating enteroviral disease among SOT recipients noted predominantly GI mediated disease, with a minority (2/11, 15%) of transplant recipients reporting neurologic symptoms relative to their non-transplant counterparts (99). In a separate case report, an adult renal transplant recipient developed acute flaccid myelitis in the context of enteroviral detection from nasopharyngeal testing, later confirmed to be due to EVD-68 (173). Another case report described 2 adult SOT recipients with diagnosis of enteroviral meningoencephalitis, presenting initially with concerns of fever and headache, though had persistent headache symptoms (1 month) after diagnosis (174). Among HCT recipients, a case report of two children presenting with fever and seizure activity post-HCT noted detection of enterovirus by PCR (one in CSF, one in sputum) (175). A separate study noted one patient post-HCT who developed rhombencephalitis due to enteroviral infection. Interestingly, this patient had a history of enteroviral CNS infection prior to transplantation while receiving B-cell depleting therapies. The individual was treated with pleconaril and IVIG prior to transplant and had proceeded to transplantation despite known enteroviral detection in the CSF (176).

The diagnosis of enteroviral disease has markedly improved with the commercial availability of PCR-based diagnostic testing modalities. Treatment of enteroviral infections, including meningoencephalitis, is largely supportive. IVIG has been utilized in cases of enteroviral meningoencephalitis for those with compromised immune systems, including malignancy (176, 177). In a small comparative study evaluating IVIG and mortality among children with enteroviral myocarditis and sepsis noted a reduced overall risk of mortality in those who received IVIG (3.8% vs. 22.8%). Notably, in those with defects in humoral immunity, use of IVIG for treatment has been employed with success (172, 178), however the overall efficacy remains unclear for treatment of CNS mediated disease. Given the favorable risk-benefit profile for IVIG administration, it is frequently employed amongst immunocompromised patients presenting with systemic enteroviral illness. Pleconaril demonstrated promise amongst a small cohort of individuals receiving the therapy on compassionate use, comprised largely of immunocompromised individuals with chronic enteroviral meningoencephalitis, with a response rate of approximately 75% (179). However, this drug currently remains unavailable for clinical use.

### Respiratory viruses: influenza virus, respiratory syncytial virus (RSV), human metapneumovirus (hMPV)

Among the most common circulating viruses during the winter months, respiratory viruses are known for substantial disease burden among immune competent and immunocompromised patients. Influenza (*Orthomyxoviridae*), RSV (*Pneumoviridae*), and hMPV (*Pneumoviridae*) predominantly cause upper and lower respiratory tract disease, spread primarily through contact or exposure to secretions of affected individuals. Neurologic complications of influenza, RSV, and hMPV have been described (11–13, 97). Influenza virus infection has resulted in a variety of neurologic presentations, including meningoencephalitis, acute necrotizing encephalopathy, myelitis, Guillain-Barre syndrome, and febrile seizures (180–182). RSV has been identified in patients with meningoencephalitis, cerebellitis, encephalitis (183, 184). hMPV has been described as cause of neurologic disease less frequently, though cases of encephalitis and febrile seizures have been reported (185). The pathophysiology of CNS disease by respiratory viruses is believed to be in part due to direct neuronal invasion, however local cytokine responses in the CNS is suspected to further drive the disease process and manifestations (97). Amongst HCT and SOT recipients, infection with Influenza virus, RSV, and hMPV is of concern given the propensity to cause severe lower respiratory tract infection with associated morbidity and mortality (186, 187). While immunocompromised HCT and SOT recipients are at risk for severe disease from influenza, RSV, and hMPV, encephalitis secondary to these pathogens is rare. A study from Zhang et al. reported on a cohort viral encephalitis in patients who underwent haploidentical HCT (11). Among 30 patients with viral encephalitis post HCT, 15 (50%) had detection of RSV in CSF, representing the most common pathogen in their cohort. The majority of patients (80%) with RSV were treated with ribavirin, and three had also received IVIG as part of therapy. The mortality rate in this cohort with RSV detection and encephalitis was approximately 15%. Encephalitis due to influenza virus and hMPV in the post-transplant setting have not otherwise been reported in literature.

### Donor derived infections, other clinically relevant neuroinvasive viral infections

This group includes those discussed above, as donor derived infection (DDI) from neuroinvasive viruses is well documented, particularly among the SOT population (188–190). In general, rates of neuroinvasive viral DDI remain low through prevention and donor screening processes (188). One important consideration is lymphocytic choriomeningitis virus (LCMV). In immunocompetent individuals, most cases are asymptomatic and self-resolving. However, among immunocompromised patients, the clinical presentation can be more fulminant in nature, with mortality rates approaching 90% among SOT recipients (191). Diagnosis is made via serologic evaluation LCMV. Treatment is primarily supportive in nature (50). Rabies virus is a rare cause of donor derived infection—in 2004, four recipients of a donor who died of unknown causes developed neurologic symptoms within 30 days of transplant, all succumbing to rabies virus CNS disease (192). Deceased individuals with meningitis, encephalitis, or flaccid paralysis of unknown or untreatable etiology should thus be deferred given the risk of transmission of neurotropic disease (189).

## Case outcomes

### Case 1

Intravenous ganciclovir was continued, and he showed a slow but continued improvement in mentation with resolution of HHV-6 viremia over the next three weeks. A repeat lumbar puncture at three weeks of therapy returned negative for HHV-6 by PCR, and a repeat brain MRI was obtained with resolution in prior abnormalities, as expected in HHV-6 meningoencephalitis; ganciclovir was discontinued and acyclovir was restarted as prophylaxis against HSV. Additionally, the patient did not incur any neurologic deficits or regression in developmental outcomes in the months following the infection.

### Case 2

His viral load peaked at >155,655 IU/ml in the context of his recently augmented immunosuppression, though eventually trended downward until becoming undetectable after approximately 2 months of therapy. The prior MRI findings of mild leptomeningeal inflammation and frontal lobe parenchymal T2 enhancement were markedly improved on repeat MRI at approximately 4 weeks of therapy. As the majority of the inflammatory process in his right eye involved peripheral retinal structures, intravitreal antiviral therapy was deferred in his case. The inflammatory changes in his eye improved with systemic antiviral therapy, though chorioretinal scarring was observed at the end of therapy. Fortunately, as this did not involve the macula, his visual acuity was largely preserved. He transitioned to valganciclovir maintenance dosing and remained on this for approximately 1 year post-transplant in consideration of his post-transplant immunosuppression and until there was evidence of CMV T-cell immune reconstitution (Viracor T-cell immunity panel). Retrospective evaluation of the donor serum revealed the donor serostatus to be CMV donor positive, and thus the presentation likely reflected donor derived CMV disease.

## Conclusion

Viral meningoencephalitis in pediatric patients who have undergone HCT or SOT is rare. Antiviral prophylaxis measures in the post-transplant period have resulted in decreased incidence of *Herpesviridae*-associated meningoencephalitis and CNS sequalae. However, *Herpesviridae*, namely HHV-6 in HCT, remain the most frequent causative agent compared to other viral etiologies. Despite non-infectious etiologies being the more frequent cause of neurologic complications post-transplant, it is imperative that when a transplant recipient presents with fever and altered mentation, prompt diagnostic work-up and empiric therapy for viral meningoencephalitis is pursued.
